# Prescription omega-3 fatty acid products containing highly purified eicosapentaenoic acid (EPA)

**DOI:** 10.1186/s12944-017-0415-8

**Published:** 2017-01-31

**Authors:** Eliot A. Brinton, R. Preston Mason

**Affiliations:** 1 Utah Foundation for Biomedical Research and the Utah Lipid Center, 419 Wakara Way, Suite 211, Salt Lake City, UT 84108 USA; 2 Department of Medicine, Cardiovascular Division, Brigham & Women’s Hospital, Harvard Medical School, Boston, MA and Elucida Research LLC, PO Box 7100, Beverly, MA 01915-6127 USA

**Keywords:** Cardiovascular disease, Cholesterol, Drug therapy, Eicosapentaenoic acid, Icosapent ethyl, Ethyl icosapentate, Hypertriglyceridemia, Inflammation, Omega-3 fatty acids, Triglycerides

## Abstract

The omega-3 fatty acid eicosapentaenoic acid (EPA) has multiple actions potentially conferring cardiovascular benefit, including lowering serum triglyceride (TG) and non-high-density lipoprotein cholesterol (non-HDL-C) levels and potentially reducing key steps in atherogenesis. Dietary supplements are a common source of omega-3 fatty acids in the US, but virtually all contain docosahexaenoic acid (DHA) in addition to EPA, and lipid effects differ between DHA and EPA. Contrary to popular belief, no over-the-counter omega-3 products are available in the US, only prescription products and dietary supplements. Among the US prescription omega-3 products, only one contains EPA exclusively (Vascepa); another closely related prescription omega-3 product also contains highly purified EPA, but is approved only in Japan and is provided in different capsule sizes. These high-purity EPA products do not raise low-density lipoprotein cholesterol (LDL-C) levels, even in patients with TG levels >500 mg/dL, in contrast to the increase in LDL-C levels with prescription omega-3 products that also contain DHA. The Japanese prescription EPA product was shown to significantly reduce major coronary events in hypercholesterolemic patients when added to statin therapy in the Japan EPA Lipid Intervention Study (JELIS). The effects of Vascepa on cardiovascular outcomes are being investigated in statin-treated patients with high TG levels in the Reduction of Cardiovascular Events With EPA-Intervention Trial (REDUCE-IT).

## Background

Eicosapentaenoic acid (EPA) is an omega-3 polyunsaturated fatty acid with a broad range of potentially beneficial cardiovascular effects [1, 2]. Chemically, EPA is designated as 20:5, n-3, indicating that it is a 20-carbon fatty acid containing 5 double bonds, with the first double bond located at the third carbon atom from the distal end of the fatty acid tail [3]. This unique chemical structure has important biological consequences. By replacing the omega-6 fatty acid arachidonic acid (AA; 20:4, n-6) in membrane phospholipids, EPA can alter the physical properties of cellular membranes. Further, its metabolism can give rise to anti-inflammatory and anti-thrombotic lipid mediators, in contrast to pro-inflammatory pro-thrombotic factors which tend to be produced from AA [1, 3–5]. Available highly purified EPA prescription products consist of the ethyl ester of EPA because this form allows preparation of omega-3 fatty acids at far greater purity than those available by other purification methods [6].

In clinical studies, EPA has been shown to lower triglyceride (TG) and non-high-density lipoprotein cholesterol (non-HDL-C) levels, as well as other key lipid/lipoprotein parameters [7, 8]. Unlike products containing the omega-3 fatty acid docosahexaenoic acid (DHA), however, EPA does not raise low-density lipoprotein cholesterol (LDL-C) levels [7–9]. Additionally, EPA has other non-lipid and non-lipoprotein effects that appear to beneficially reduce multiple steps in atherogenesis [10], including protecting against oxidative damage [5, 11–13], improving vascular and endothelial function [14–16], inhibiting monocyte movement into early lesions and subsequent conversion to macrophages and foam cells [17–19], modulating inflammation [20, 21], supporting anti-oxidant and anti-inflammatory functions of high-density lipoprotein (HDL) [22], promoting HDL-mediated cholesterol efflux from macrophages [22], reducing atherosclerotic plaque formation, progression, and vulnerability to rupture [18, 23–27], and decreasing platelet-mediated thrombus formation [28, 29]. EPA also may reduce blood pressure, likely attributable to improvement of endothelial function [30]. Importantly, many of these effects have been observed with EPA alone or are additive to those of statins. In contrast, EPA’s effects on arrhythmias are complex and equivocal [30].

Lipid guidelines and recommendations vary regarding the suggested use of omega-3 fatty acids in general and, when mentioned, EPA in particular. For example, the National Lipid Association (NLA) recommends omega-3 fatty acids as a first-line option for patients with very high TG levels (≥500 mg/dL) and as an add-on option to statin therapy for those with high TG levels (200–499 mg/dL) in order to achieve specific lipid goals, but notes that omega-3 fatty acid drugs may raise LDL-C levels if they contain DHA [31]. The American College of Cardiology/American Heart Association (ACC/AHA) cholesterol treatment guidelines note in passing the efficacy of omega-3 fatty acids in reducing TG levels in patients with severe hypertriglyceridemia [32] but focus on diet and lifestyle measures, and contain very little about prescription omega-3 agents in particular, or on TG-lowering medications in general. The Japan Atherosclerosis Society (JAS) indicates that the addition of EPA to a statin can be useful for treating high-risk patients with LDL-C levels ≥140 mg/dL [33].

Most prescription omega-3 products approved in the US have both EPA and DHA, as do virtually all marketed dietary supplements. Only two prescription formulations of highly purified EPA (without DHA) are currently approved in the world: Epadel® in Japan (ethyl icosapentate; Mochida Pharmaceuticals Co, Ltd, Tokyo, Japan) and Vascepa® in the US (icosapent ethyl; Amarin Pharma Inc., Bedminster, NJ, USA). This review examines key safety and efficacy considerations of these two highly purified prescription EPA products.

## Prescription EPA products

Epadel was approved in Japan more than 2 decades ago, and is indicated for the treatment of hyperlipidemia as well as improvement of ulcer, pain, and cold feeling associated with arteriosclerosis obliterans [34]. Vascepa was approved in the US in 2012, and is indicated as an adjunct to diet to reduce TG levels in adults with severe hypertriglyceridemia (≥500 mg/dL) [35]. Both are composed of highly purified EPA ethyl ester (Table 1) [7, 34–36]; however, their purification processes differ slightly and their capsule size and dosing recommendations differ. Epadel (approved only in Japan) is usually administered at a dose of 1.8 g/day (0.6 g tid after food) and can be increased to 2.7 g/day if TG levels remain abnormal [34]. Epadel is available as 300-mg, 600-mg, and 900-mg capsules to be taken two or three times daily after meals to achieve the desired daily dose [34]. Vascepa (approved only in the US) is manufactured as 500-mg and 1000-mg capsules and is approved at a dose of 4 g/day, to be given as two 1000-mg capsules, or four 500-mg capsules, twice daily with food [35].

**Table 1:** Prescription EPA Products [34, 35]

	Epadel	Vascepa
Year approved	1988	2012
Country	Japan	USA
Generic name	Ethyl icosapentate; icosapent	Icosapent ethyl
Structure		
Molecular formula	C_22_H_34_O_2_	C_22_H_34_O_2_
Molecular weight	330.50	330.51
Indications	Hyperlipidemia; improvement of ulcer, pain and cold feeling associated with arteriosclerosis obliterans	Adjunct to diet to reduce TG levels in adults with severe (≥500 mg/dL) hypertriglyceridemia
Formulation	300-mg, 600-mg, or 900-mg capsules	500-mg or 1000-mg capsules
Dosage and administration	1.8 g/day^a^ (twice daily after meals)	4 g/day (twice daily with food)

^a^Recommended dosage for treatment of hyperlipidemia; dose may be increased to 1 capsule three times per day if TG levels are abnormal. *EPA* eicosapentaenoic acid, *TG*, triglyceride

## Safety and tolerability of prescription EPA products

In JELIS, adverse events (AEs) that were more common in the Epadel group than the control group were, respectively, gastrointestinal disturbance (nausea, diarrhea, or epigastric discomfort; 3.8 vs 1.7%; *P* <0.0001), skin abnormality (eruption, itching, exanthema, or eczema; 1.7 vs 0.7%; *P* <0.0001), hemorrhage (cerebral, fundal, epistaxis, and subcutaneous; 1.1 vs 0.6%; *P* = 0.0006), and increased glutamic oxaloacetic transaminase (0.6 vs 0.4%; *P* = 0.03) [36]. Beyond JELIS, the safety of Epadel has been followed for more than 15 years in spontaneous reporting in post-marketing regulatory safety surveillance, as well as in several randomized clinical trials in Japan. It has been shown to have very good safety and tolerability, with AEs reported in 665 of 15,081 patients (4.4%) [34]. The most common AEs were nausea (0.44%) and abdominal discomfort (0.32%). Vascepa has also been shown to be safe and well tolerated, with a safety profile similar to placebo in patients with hypertriglyceridemia in the Multi-center, Placebo-controlled, Randomized, Double-blind, 12-week Study with an Open-label Extension (MARINE) and ANCHOR studies [7, 8]. In a pooled analysis of Vascepa clinical trials, arthralgia was the only AE that occurred in >2% of patients and with an incidence greater than placebo (14/622 [2.3%] Vascepa patients vs 3/309 [1.0%] placebo patients) [35]. In addition, no drug-drug interactions between Vascepa and atorvastatin, warfarin, rosiglitazone, or omeprazole were observed in pharmacokinetic studies [37–40].

Omega-3 fatty acids tend to reduce platelet activity, which might contribute to their potential cardiovascular benefits [41], but which might also lead to excess bleeding. Some studies of omega-3 fatty acids have documented a prolongation of bleeding time, although not exceeding normal limits [34, 35]. Further, an aggregate endpoint of various types of hemorrhage was increased slightly with Epadel in the Japan EPA Lipid Intervention Study (JELIS) [36]. It is recommended that patients receiving treatment with Vascepa or Epadel should be monitored periodically on a clinical basis only (without any recommended testing) when either of these drugs is added to medications that affect coagulation [34, 35]. Such monitoring does not differ, however, from that recommended for such patients not taking EPA. Importantly, omega-3 fatty acids do not appear to increase the risk of clinically significant bleeding when used alone or in combination with antiplatelet or antithrombotic medications [41–43].

## Efficacy of prescription EPA products

### Epadel

In clinical studies of Japanese patients with elevated TG levels, Epadel reduced serum TG levels by 14 to 20% and total cholesterol levels by 3 to 6%, with stable effects from 24 to 52 weeks [34]. The magnitude of the TG effects was related to baseline TG values in that TG decreases of −23, −103, and −172 mg/dL were noted with baseline levels of 150–299 mg/dL, 300–599 mg/dL, and ≥600 mg/dL, respectively. Similarly, decreases in serum cholesterol were proportional to baseline levels, as decreases of −6, −18, and −28 mg/dL occurred in patients with baseline levels of 220–259 mg/dL, 260–299 mg/dL, and ≥300 mg/dL, respectively.

The effect of Epadel on cardiovascular outcomes was investigated in JELIS, which was a prospective, randomized, open-label, blinded endpoint evaluation (PROBE) trial that enrolled 18,645 hypercholesterolemic Japanese patients (baseline total cholesterol ≥250 mg/dL and LDL-C ≥170 mg/dL) with or without coronary artery disease (CAD) [36, 44]. Patients received pravastatin 10 mg or simvastatin 5 mg daily as first-line treatment, which was up-titrated as needed to achieve goal LDL-C levels, as recommended by Japanese guidelines [36, 44]. Patients were then randomized to receive Epadel (1.8 g/day as 0.6 g three times daily) or no additional treatment (control group). The primary endpoint was any major coronary event, consisting of sudden cardiac death, myocardial infarction (MI), unstable angina pectoris, angioplasty, stenting, or coronary artery bypass grafting [36].

Among JELIS patients, 14,981 had no CAD at baseline (primary prevention cohort) and 3664 had documented CAD (secondary prevention cohort) [36]. At baseline, the mean total cholesterol and LDL-C levels were 274 and 181 mg/dL, respectively, and the median TG level was 153 mg/dL [36]. Upon study entry, patients received statin therapy up-titrated to achieve cholesterol goals per Japanese guidelines, consisting of pravastatin or simvastatin at mean daily doses of 10.0 and 5.6 mg, respectively. In contrast to the studies noted above, Epadel had only modest TG-lowering effects (9% in the EPA group vs 4% in the control group, a difference of approximately 5%), without changes in other lipid levels. After a mean follow-up of 4.6 years, Epadel significantly reduced risk of major coronary events by 19% compared with control (hazard ratio [HR], 0.81; 95% confidence interval [CI], 0.69–0.95; *P* = 0.011) [36]. A very important but largely unrecognized fact about JELIS is that it was the first randomized clinical trial to show additional cardiovascular benefit of any medication added to a statin. A statistically significant risk reduction for major coronary events with Epadel was also seen in the secondary prevention cohort alone (HR, 0.81; 95% CI, 0.657–0.998; *P* = 0.048), and a similar trend, albeit not statistically significant, was seen in the primary prevention cohort alone (HR, 0.82; 95% CI, 0.63–1.06; *P* = 0.132). Of the secondary endpoints evaluated, Epadel significantly reduced risk of unstable angina in the entire study population (HR, 0.76; *P* = 0.014) and in the secondary prevention cohort (HR, 0.72; *P* = 0.019) [36]. Although the largest numbers of patients with primary endpoint events had “soft” endpoints of unstable angina (*n* = 340) or revascularization (*n* = 413), which may have resulted from bias due to the open-label nature of the trial, it is important to note that primary prevention for two composites of “hard” endpoints, coronary death or MI, and fatal or nonfatal MI, were decreased by 22 and 23%, respectively, comparable to that of the primary composite endpoint of 19% [36]. This strongly suggests that the positive finding of cardiovascular disease event reduction in JELIS was not an artifact of the open-label trial design.

Substudies of JELIS explored the relationship between patient types and cardiovascular disease benefit from Epadel therapy (Table 2). In the primary prevention cohort, 957 patients had TG levels ≥150 mg/dL and high-density lipoprotein cholesterol (HDL-C) levels <40 mg/dL at baseline. These patients exhibited an elevated risk of developing CAD compared with those without these abnormalities, and Epadel greatly reduced the risk of major coronary events in this high-risk subgroup by 53% compared with the control group (HR, 0.47; 95% CI, 0.23–0.98; *P* = 0.043) [45]. Surprisingly, the lipid effects of EPA were also very modest in this subgroup (as in the overall study population) despite the higher baseline TG level (median 272 mg/dL), with TG level decreases of 23 versus 18% in the Epadel and control groups, respectively. In a somewhat related finding, in a cohort of 4565 JELIS patients with impaired glucose metabolism (defined as fasting blood glucose ≥110 mg/dL, diabetes, or use of antidiabetic drugs), Epadel significantly reduced the risk of major coronary events by 22% compared with control treatment (HR, 0.78; 95% CI, 0.60–0.998; *P* = 0.048) [46]. Epadel was also similarly effective in reducing the risk of major coronary events in other high-risk subgroups, including patients with prior peripheral artery disease (56%; HR, 0.44; 95% CI, 0.19–0.97) [47], not achieving LDL-C and HDL-C goals (38%; HR, 0.62; 95% CI, 0.43–0.88) [48], and with either prior MI (27%; HR, 0.73; 95% CI, 0.54–0.98) or prior coronary intervention (35%; HR, 0.65; 95% CI, 0.48–0.89) [49]. Epadel also decreased recurrent stroke by 20% (HR, 0.80; 95% CI, 0.64–0.997; *P* = 0.047) in the secondary prevention subgroup [50]. In an analysis of patient adherence in JELIS, EPA conferred substantial risk reduction in sudden cardiac death and fatal/non-fatal MI compared with statin alone (HR, 0.55; 95% CI, 0.34–0.88; *P* = 0.014) in the secondary prevention population of patients with good adherence to EPA plus statin therapy [51]. As expected, analyses of fatty acids in patients from JELIS found that intervention with EPA led to an increase in both plasma EPA concentration and the EPA/AA ratio. Importantly, the risk of major coronary events (primary study endpoint) was reduced proportionally to the increases in plasma EPA concentration and EPA/AA ratio [52].

**Table 2:** Effect of Epadel on Risk of Major Coronary Events^a^ in JELIS and JELIS Substudies

Analysis Group	Cohort	N	HR (95% CI)	*P* Value	Reference
JELIS	All patients	18,645	0.81 (0.69–0.95)	0.011	Yokoyama, 2007 [36]
Impaired glucose metabolism^b^	All patients	4565	0.78 (0.60–0.998)	0.048	Oikawa, 2009 [46]
Peripheral artery disease	All patients	223	0.44 (0.19–0.97)	0.041	Ishikawa, 2010 [47]
JELIS	1° prevention	14,981	0.82 (0.63–1.06)	0.132	Yokoyama, 2007 [36]
TG ≥150 mg/dL and/or HDL-C <40 mg/dL	1° prevention	957	0.47 (0.23–0.98)	0.043	Saito, 2008 [45]
Patients not achieving LDL-C and non-HDL-C goals^c^	1° prevention	6592	0.62 (0.43–0.88)	0.007	Sasaki, 2012 [48]
JELIS	2° prevention	3664	0.81 (0.657–0.998)	0.048	Yokoyama, 2007 [36]
Prior myocardial infarction	2° prevention	1050	0.73 (0.54–0.98)	0.033	Matsuzaki, 2009 [49]
Prior coronary intervention	2° prevention	895	0.65 (0.48–0.89)	0.007	Matsuzaki, 2009 [49]

*CI* confidence interval, *HDL-C* high-density lipoprotein cholesterol, *HR* hazard ratio, *JELIS* Japan EPA Lipid Intervention Study, *LDL-C* low-density lipoprotein cholesterol, *non-HDL-C* non-high-density lipoprotein cholesterol, *TG* triglyceride

^a^The primary endpoint, major coronary events, consisted of sudden cardiac death, myocardial infarction, unstable angina pectoris, angioplasty, stenting, or coronary artery bypass grafting [36]

^b^Defined as fasting plasma glucose ≥110 mg/dL at study registration or after 6 months, physician-diagnosed diabetes mellitus, or use of antidiabetic drugs within first year of study [46]

^c^LDL-C goal as recommended in 2007 Japanese Atherosclerosis Society guidelines and a non-HDL-C goal of 30 mg/dL higher than the LDL-C goal as recommended in Adult Treatment Panel III guidelines [48]

Two large ongoing clinical trials are evaluating EPA-only therapy in an effort to confirm the results of JELIS. One trial is examining the effects of EPA added to a statin on the incidence of cardiovascular events in patients with CAD. The EPA being used is Epadel and the trial is titled the Randomized Trial for Evaluation in Secondary Prevention Efficacy of Combination Therapy-Statin and Eicosapentaenoic Acid (RESPECT-EPA) [53]. The primary endpoints include a composite of CAD including sudden cardiac death, MI, revascularization, and hospitalization for unstable angina and a composite of cerebrovascular disorders including fatal and non-fatal stroke. The other trial is testing Vascepa and is detailed in the next section.

Recently, the Combination Therapy of Eicosapentaenoic Acid and Pitavastatin for Coronary Plaque Regression Evaluated by Integrated Backscatter Intravascular Ultrasonography (CHERRY) study investigated the effects of Epadel 1.8 g/day with pitavastatin 4 mg/day versus pitavastatin alone on the progression of coronary plaque via integrated backscatter intravascular ultrasound in approximately 200 patients [54, 55]. After 6 – 8 months, total plaque volume and volume of the lipid-rich portion of the plaques were significantly decreased in the EPA arm after adjustment for confounding factors. Specifically, the percentage of patients with plaque regression was significantly higher with EPA versus without EPA (50 vs 24%, respectively; *P* <0.001) [55]. The change in the EPA/AA ratio, which was, of course, significantly increased in the group receiving EPA, correlated significantly and inversely with the change in plaque volume (r = −0.332; *P* <0.001). These improvements in coronary atherosclerosis corroborate the decrease in cardiovascular events in JELIS and suggest that EPA may reduce the residual cardiovascular risk in patients with prior CHD who are already on moderate-intensity statin treatment.

### Vascepa

**Fig. 1:**
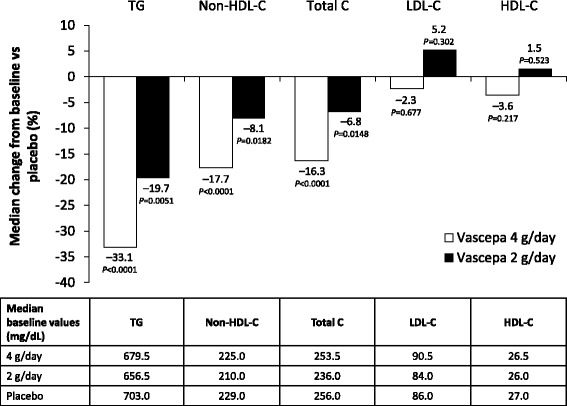
Effect of Vascepa on lipid levels in patients with very high TG levels (≥500 and ≤2000 mg/dL) in the MARINE study. Shown are the median changes from baseline to week 12 in the intent-to-treat population [7]. HDL-C, high-density lipoprotein cholesterol; LDL-C, low-density lipoprotein cholesterol; MARINE, Multi-center, Placebo-controlled, Randomized, Double-blind, 12-week Study with an Open-label Extension; Non-HDL-C, non-high-density lipoprotein cholesterol; TG, triglyceride; Total C, total cholesterol

**Fig. 2:**
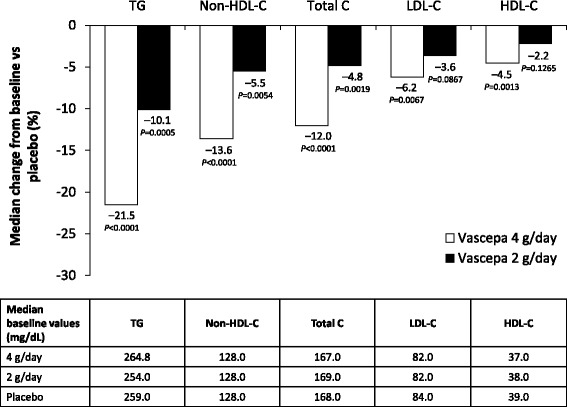
Effect of Vascepa on lipid levels in patients with high TG levels (≥200 and <500 mg/dL) despite LDL-C control while on statin therapy in the ANCHOR study. Shown are the median changes from baseline to week 12 in the intent-to-treat population [8]. HDL-C, high-density lipoprotein cholesterol; LDL-C, low-density lipoprotein cholesterol; Non-HDL-C, non-high-density lipoprotein cholesterol; TG, triglyceride; Total C, total cholesterol

The efficacy of Vascepa in modulating lipid levels was demonstrated in 2 randomized, double-blind, placebo-controlled, multicenter phase 3 studies known as MARINE and ANCHOR [7, 8]. In both studies, patients entered a 4–6-week stabilization period during which they were provided dietary instructions and discontinued any other TG-lowering therapy such as fibrates, niacin, or omega-3 fatty acids. Treatment with stable doses of a statin with or without ezetimibe was allowed in MARINE and was required in ANCHOR. After the stabilization period in MARINE, 229 patients with very high TG levels (≥500 and ≤2000 mg/dL) were randomized to treatment with Vascepa 4 g/day, Vascepa 2 g/day, or placebo for 12 weeks [7]. Both doses of Vascepa significantly reduced TG levels. Compared with placebo, the median change in TG levels from baseline was −33.1% (*P* <0.0001) with the 4 g/day dose and −19.7% (*P* = 0.0051) with the 2 g/day dose. Both doses of Vascepa also significantly reduced non-HDL-C and total cholesterol levels compared with placebo, but did not significantly affect LDL-C or HDL-C levels (Fig. 1). In ANCHOR, 702 patients with high TG levels (≥200 and <500 mg/dL) and optimal LDL-C levels (≥40 and <100 mg/dL) on statin therapy were randomized to treatment with Vascepa 4 g/day, Vascepa 2 g/day, or placebo for 12 weeks [8]. Again, compared with placebo, both doses of Vascepa significantly reduced TG levels, with a median change from baseline of −21.5% for the 4 g/day dose (*P* <0.0001) and −10.1% for the 2 g/day dose (*P* = 0.0005). Compared with placebo, both doses of Vascepa significantly reduced non-HDL-C and total cholesterol levels, and the 4 g/day dose produced small but statistically significant reductions in LDL-C and HDL-C levels (Fig. 2).

Further laboratory testing in MARINE and ANCHOR revealed that Vascepa has additional, and apparently beneficial, effects. Vascepa 4 g/day significantly reduced circulating markers of inflammation and oxidation, including high-sensitivity C-reactive protein by −36% in MARINE (*P* <0.01) and −22% in ANCHOR (*P* <0.001), lipoprotein-associated phospholipase A_2_ by −14% in MARINE (*P* <0.001) and −19% in ANCHOR (*P* <0.0001), and oxidized low-density lipoprotein (LDL) by −13% in ANCHOR (*P* <0.0001) compared with placebo [21]. Importantly, Vascepa 4 g/day did not worsen glycemic control in the subset of ANCHOR patients with type 2 diabetes compared with placebo [56]. Interestingly, the effects of Vascepa appeared numerically more pronounced in patients with less-controlled diabetes (hemoglobin A_1c_ greater than the median value of 6.8%) than in those with better-controlled diabetes [56]. As expected, treatment with Vascepa 4 g/day and 2 g/day in MARINE and ANCHOR led to significant increases in plasma EPA (see subsequent section on plasma EPA levels) and corresponding decreases in the AA/EPA ratio compared with placebo (*P* <0.0001 for all comparisons) [57, 58].

Lipoprotein content and lipoprotein particle concentration and size are additional factors that may influence atherogenicity. Levels of apolipoprotein B (apoB) were significantly reduced with Vascepa 4 g/day in MARINE (*P* = 0.0019) and ANCHOR (*P* <0.0001) [7, 8]. In MARINE, nuclear magnetic resonance analyses (LipoScience, Inc. LipoProfile) showed that Vascepa 4 g/day significantly reduced particle concentrations of large very-low-density lipoprotein cholesterol (VLDL) (*P* = 0.0211), total LDL (*P* = 0.0006), small LDL (*P* <0.0001), and total HDL (*P* = 0.0063), as well as VLDL particle size (*P* = 0.0017) compared with placebo [59]. Similar results were observed in ANCHOR, where Vascepa 4 g/day significantly reduced particle concentrations of total VLDL (*P* = 0.0002), LDL (*P* = 0.0017), and HDL (*P* <0.0001) and large VLDL, small LDL, and large HDL (all *P* <0.0001), and caused corresponding changes in lipoprotein particle sizes [60]. The reduction in LDL particle concentration with pure EPA is a novel finding among prescription omega-3 fatty acids, and is consistent with data showing that Vascepa significantly lowers apoB [59, 60]. Vascepa also is reported to reduce remnant-like particle cholesterol (RLP-C) levels in MARINE and ANCHOR [61] using an immunoseparation assay [62–64]. Remnant lipoproteins are partially catabolized TG-rich lipoproteins, are known to be atherogenic in the relatively uncommon situation of dysbetalipoproteinemia, and are thought to be a risk factor for atherosclerotic cardiovascular disease in the general population. Although some recent publications have presented calculated remnant cholesterol levels (obtained from a basic lipid panel by subtracting calculated LDL-C from non-HDL-C) as a readily obtainable surrogate for measured RLP-C levels [65], it should be noted that such a calculation is the same as simply dividing the measured plasma or serum TG level by 5 and thus does not contribute uniquely to our understanding of remnant levels. Finally, Vascepa 4 g/day significantly reduced apolipoprotein C-III (apoC-III) levels compared with placebo in MARINE and ANCHOR (both *P* <0.0001) [66]. ApoC-III impairs TG lipolysis and hepatic uptake of TG-rich lipoproteins, is strongly and positively related to levels of remnant lipoproteins, and appears to promote atherogenesis by these and other mechanisms [67].

To investigate potential direct antioxidant mechanisms by which Vascepa may lower circulating levels of apoB-containing atherogenic lipoprotein particles, an in vitro assay assessed the antioxidant effects of EPA on small dense LDL, LDL, and VLDL isolated from human plasma [13]. EPA was found to inhibit the oxidation of small dense LDL and LDL in a dose-dependent manner and also inhibited VLDL oxidation with higher potency than the other lipoprotein species [13]. Notably, this inhibition of particle oxidation was enhanced when EPA was combined with the active metabolite of atorvastatin, while DHA caused less inhibition of oxidation of the apoB-containing particles than did EPA [13].

A large cardiovascular outcome trial of Vascepa is now well underway. It is examining the effects of EPA on cardiovascular outcomes in high-risk patients with persistent hypertriglyceridemia despite statin therapy and either established or high cardiovascular disease risk. The trial is titled the Reduction of Cardiovascular Events With EPA–Intervention Trial (REDUCE-IT; NCT01492361) [68]. Patients with a fasting TG level ≥150 mg/dL (or ≥200 mg/dL, depending on the phase of trial recruitment) are being randomized to Vascepa 4 g/day or placebo, and the primary efficacy endpoint is a composite of cardiovascular death, MI, stroke, revascularization, and hospitalization for unstable angina. The study is anticipated to be completed in late 2017 with results available in 2018. It should be noted that Vascepa is not approved by the US FDA to reduce the risk of coronary heart disease; the effect of Vascepa on the risk of cardiovascular mortality and morbidity has not been determined.

## Prescription EPA-only products versus other omega-3 fatty acid products

Vascepa is the only highly purified EPA prescription product approved in the US. Other products, including other prescription omega-3 fatty acid formulations [69–71] and dietary supplements [72, 73], contain substantial amounts of DHA in addition to EPA. DHA (22:6, n-3) is structurally and chemically distinct from EPA, and although DHA can be derived metabolically from EPA, very little, if any, of the reverse conversion (DHA to EPA) occurs in vivo [3, 74]. Importantly, products containing DHA have been shown to raise LDL-C levels, which may attenuate its other lipid benefits [9, 70, 71, 75–77]. In contrast, products with EPA but lacking DHA do not raise LDL-C levels (compared with placebo or control), as noted with Vascepa in MARINE and ANCHOR and with Epadel in JELIS in certain patient populations [7, 8, 36].

These differential effects of EPA versus DHA on LDL-C levels may be attributable to one or more of at least three mechanisms. First, DHA (but not EPA) can downregulate expression of the LDL receptor and receptor-mediated clearance of LDL-C by the liver, which is an important inverse regulator of LDL-C levels. This appears to occur via a suppression of sterol regulatory element binding protein (SREBP)-2 expression by DHA [78, 79]. Second, DHA can upregulate activity of cholesteryl ester transfer protein (CETP), which promotes core lipid transfer among lipoproteins, tending to increase LDL-C and decrease HDL-C levels, while EPA has a neutral effect on CETP activity [79]. Third, DHA may exceed EPA in its upregulation of lipoprotein lipase activity, which would increase conversion of VLDL to LDL, thus tending to increase LDL-C [9, 76].

### Omega-3 fatty acid dietary supplements

The many non-prescription fish oil products available in the US are not over-the-counter (OTC) drugs; rather, they are dietary supplements [80]. This is an important distinction because dietary supplements are not subject to the same regulatory oversight and testing as prescription drugs or OTC drugs [81–84]. They are considered safe until proven otherwise [83], but are not considered appropriate for treatment of disease and thus are not considered appropriate substitutes for prescription products [80]. Further, omega-3 fatty acid dietary supplements tend not only to have less EPA and DHA per gram than prescription products, but there are qualitative and potential safety differences as well [80]. The amounts of omega-3 fatty acids actually present in dietary supplements may vary widely and are usually substantially below the content stated on the label [73, 85–88]. Of greater potential importance, the non-omega-3 components of dietary supplement fish oil products usually include large amounts of saturated fatty acids [89, 90]. Of greatest significance, most dietary supplement omega-3 preparations are oxidized above the maximum levels recommended by industry standards [86, 88, 91–94]. A recent study of six leading dietary supplements (based on sales) examined each for purity, omega-3 fatty acid levels, and oxidation effects [94]. Omega-3 fatty acid content varied widely, from 33 to 79%, and the remaining non-omega-3 fat included more than 30 different fatty acid species. Further, primary and total peroxide levels exceeded international thresholds. If attempting to reach the recommended prescription dose of 4 g/day of omega-3 fatty acids, the approximate 13 capsules (based on the average 300 mg/capsule) required would lead to intake of excess calories and high intake of various unwanted fats, including oxidized fats, potentially negating any therapeutic benefits [94]. By contrast, Vascepa was found to be without elevations in lipid oxidation products [94]. Interestingly, oxidation of small dense LDL in vitro was found to be inhibited by >95% (*P* <0.001) with non-oxidized forms of omega-3 fatty acids, but not with a combination of oxidized and non-oxidized omega-3 fatty acids isolated from dietary supplements and then added to the in vitro assay [94]. These data suggest a mechanism by which at least some of the potential benefits of non-oxidized omega-3 fatty acids (such as prescription products) may be absent from oxidized preparations of omega-3 fatty acids (such as dietary supplements). The human health effects of partially oxidized dietary supplements are not known, although the levels of oxidized lipids have been shown to be predictive of clinical events in patients with CAD [94–96]. In addition to its direct antioxidant properties, pure EPA has also been shown to inhibit the oxidation-induced changes in membrane structural organization such as the formation of membrane-restricted cholesterol crystalline domains [5]. Although both EPA and DHA readily intercalate into the phospholipid bilayers which constitute the outer membranes of cells, EPA can provide potentially important in situ antioxidant effects and can stabilize cell membranes in the presence of increasing unesterified cholesterol content. In contrast, DHA tends to increase membrane fluidity, reduce overall membrane thickness/width, and promote cholesterol crystalline domain formation. These differences suggest that EPA may have net atheroprotective effects on cell membranes under disease-like conditions not available with DHA [97].

## Plasma EPA levels with EPA treatment

**Fig. 3:**
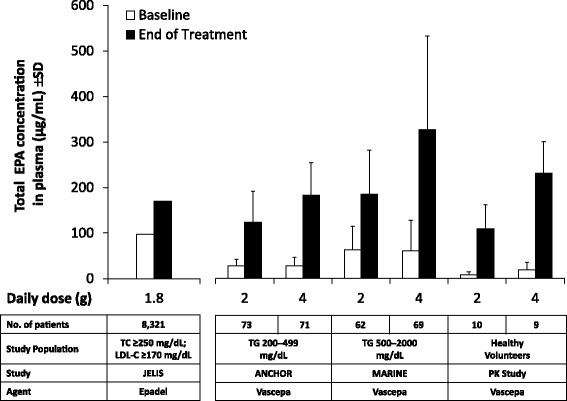
Mean trough total EPA concentrations (±SD) in plasma at baseline and at EOT in Vascepa and Epadel studies [98]. The PK study examined Vascepa in healthy adult subjects [101]. MARINE evaluated Vascepa in patients with very high TG levels (≥500 and ≤2000 mg/dL) [7]. ANCHOR evaluated Vascepa in patients with high TG levels (≥200 and <500 mg/dL) despite LDL-C control while on statin therapy [8]. Finally, JELIS evaluated Epadel in Japanese patients with hypercholesterolemia (total cholesterol ≥250 mg/dL) with or without CAD [36]. EOT = 4 weeks for the phase 1 PK study and 12 weeks in the MARINE and ANCHOR studies. JELIS was an outcome study with a planned follow-up of 5 years. Baseline values were not subtracted from EOT values. CAD, coronary artery disease; EOT, end of treatment; EPA, eicosapentaenoic acid; JELIS, Japan EPA Lipid Intervention Study; LDL-C, low-density lipoprotein cholesterol; MARINE, Multi-center, Placebo-controlled, Randomized, Double-blind, 12-week Study with an Open-label Extension; PK, pharmacokinetic; SD, standard deviation; TG, triglyceride

Dietary fish intake raises plasma EPA levels, and therefore the Japanese patients in JELIS, with their customary high fish intake diet, had higher baseline EPA levels than did patients from Western countries in MARINE and ANCHOR [36, 98]. Interestingly, treatment with 4 g/day pure EPA (Vascepa) in MARINE and ANCHOR resulted in on-treatment EPA levels similar to or higher than the levels in Japanese patients treated with 1.8 g/day pure EPA (Epadel) in JELIS (Fig. 3) [98], and comparable to the higher EPA levels (≥150 μg/mL) associated with a significantly lower risk of major coronary events in JELIS versus patients with lower EPA levels (<87 μg/mL; *P* = 0.042) [52].

## Discussion

JELIS had several strengths, including its large sample size (*N* = 18,645) and long follow-up (mean 4.6 years), and the study showed a statistically significant and clinically significant 19% decrease in its primary endpoint of major coronary events [36]. Several limitations of the study should be considered, however. Recruitment was based on the presence of hypercholesterolemia (>250 mg/dL) with or without CAD, rather than hypertriglyceridemia, which is the usual indication for omega-3 treatment. JELIS patients had relatively low baseline TG levels (mean 153 mg/dL), which may account for the finding of only a very small (5%) reduction versus placebo in plasma TG levels with Epadel treatment (although this was highly statistically significant, *P* <0.0001) [36]. The finding of a 19% decrease in CHD in conjunction with such a small TG change, and no other significant lipid changes, suggests that EPA may have cardiovascular effects beyond its lipid/lipoprotein effects. A recent study of EPA treatment that examined fatal and nonfatal cardiovascular events 1 year after percutaneous coronary intervention also demonstrated cardiovascular benefit but minimal lipid effects [99]. Patients with acute coronary syndrome (*N* = 241) were randomized to pitavastatin alone or pitavastatin plus EPA. There was an absolute risk reduction of 11% with EPA treatment (HR, 0.42; 95% CI, 0.21–0.87; *P* = 0.02) despite the fact that TG levels remained the same and were comparable for both groups (*P* = 0.61) and LDL-C reduction was comparable for both groups (*P* = 0.21) during the study period [99]. Anti-atherogenic effects of EPA that may be independent of lipid changes include actions on key steps in atherogenesis, including inflammation, thrombosis, and membrane cholesterol metabolism, as noted above [10].

Another limitation is that the statin doses in JELIS (pravastatin 10 mg or simvastatin 5 mg) were low, at least by Western standards. The doses were, however, mandated to be up-titrated as needed to achieve LDL-C goals from Japanese Ministry of Health, Labor, and Welfare guidelines [36, 44]. Importantly, the modest pravastatin dose used in JELIS was shown to reduce cardiovascular disease events (vs control) in the Management of Elevated Cholesterol in the Primary Prevention Group of Adult Japanese (MEGA) study [100], which was cited in the 2013 ACC/AHA cholesterol treatment guidelines in support of the recommendation for use of statins in an appropriate primary prevention population [32]. Thus, there is precedent for extrapolating results from a Japanese-only population to US treatment situations, including lipid guidelines for reducing cardiovascular risk [32]. Finally, while JELIS lacked a blinded control group, the blinded endpoint evaluation (ie, PROBE design) adds some confidence to conclusions based on the study findings. This is especially true since the “hard endpoints” (such as blindly adjudicated MI), not likely to be affected by unblinding of patients and study staff, were reduced to a similar degree as the primary composite endpoint.

Although robust randomized controlled trial evidence for decreased cardiovascular disease events from EPA is based at present on only one large trial (JELIS), there is now corroboration of this finding from in vitro mechanistic studies [13], decreased atherosclerosis endpoints in two trials [7, 8], and decreased clinical cardiovascular disease events in a recent additional trial [99]. Further, two key findings from JELIS subanalyses and substudies suggest that the REDUCE-IT trial of EPA may also show cardiovascular disease benefit. First cardiovascular disease reduction in JELIS was proportional to on-treatment EPA levels, and the higher dose of EPA in REDUCE-IT vs JELIS (4 vs 1.8 g/d, respectively) suggests that REDUCE-IT may show greater cardiovascular disease reduction. Second, in JELIS, cardiovascular disease reduction was greater in the subgroup of patients with higher baseline TG and lower baseline HDL-C levels, and REDUCE-IT subjects have all been recruited prospectively for and enrolled based on higher baseline TG levels. Also, the double-blind, placebo-controlled design of REDUCE-IT may add greater confidence to study findings compared with JELIS, even though the patient sample size is less than half.

## Conclusions

EPA has several potentially beneficial cardiovascular effects, including reducing TG levels and other markers or factors of atherogenesis without raising LDL-C levels, as does DHA. This is supported by NLA recommendations which endorse prescription omega-3 fatty acid products for hypertriglyceridemia but caution that products containing DHA may raise LDL-C, which may interfere with achievement of LDL-C treatment goals. In addition, purified EPA, but not DHA, has been demonstrated to have potentially atheroprotective antioxidant and membrane-stabilizing effects.

Not only do fish oil omega-3 fatty acid dietary supplements all contain DHA, but as dietary supplements they are not as highly regulated as prescription or OTC drugs. Their content of EPA and DHA can be substantially lower than the amounts stated on the product labels. In addition, omega-3 dietary supplements usually contain significant amounts of saturated fats and oxidation products, which may interfere with their intended therapeutic benefits. For these reasons, dietary supplements are not intended for the treatment of diseases, such as hypertriglyceridemia.

Epadel and Vascepa are prescription products containing highly purified EPA ethyl esters. Epadel reduced cardiovascular events when added to statins in JELIS, has been associated with reduced atherosclerotic plaque, and has a long history of use in Japan with a favorable safety and tolerability profile. Vascepa is supported by safety and efficacy data from two prospective phase 3 trials in patients with either high or very high TG levels, by the safety profile of several years of post-marketing clinical use in the US, and by the ongoing collection of safety and efficacy data over several years in REDUCE-IT. The effects of Epadel on cardiovascular outcomes in a secondary prevention population are currently undergoing confirmation in the RESPECT-EPA trial, while the cardiovascular disease effects of Vascepa in patients with persistently high TG levels despite statin use are under investigation in REDUCE-IT.

## Abbreviations

AA: Arachidonic acid
ACC/AHA: American College of Cardiology/American Heart Association
AEs: Adverse events
apoB: Apolipoprotein B
apoC-III: Apolipoprotein C-III
CAD: Coronary artery disease
CHD: Coronary heart disease
CHERRY: Combination Therapy of Eicosapentaenoic Acid and Pitavastatin for Coronary Plaque Regression Evaluated by Integrated Backscatter Intravascular Ultrasonography study
CI: Confidence interval
DHA: Docosahexaenoic acid
EPA: Eicosapentaenoic acid
HDL: High-density lipoprotein
HDL-C: High-density lipoprotein cholesterol
HR: Hazard ratio
JAS: Japan Atherosclerosis Society
JELIS: Japan EPA Lipid Intervention Study
LDL: Low-density lipoprotein
LDL-C: Low-density lipoprotein cholesterol
MARINE: Multi-center, Placebo-controlled, Randomized, Double-blind, 12-week Study with an Open-label Extension
MEGA: Management of Elevated Cholesterol in the Primary Prevention Group of Adult Japanese study
MI: Myocardial infarction
NLA: National Lipid Association
non-HDL-C: Non-high-density lipoprotein cholesterol
OTC: Over the counter
PROBE: Prospective, Randomized, Open-label, Blinded Endpoint
REDUCE-IT: Reduction of Cardiovascular Events with EPA–Intervention Trial
RESPECT-EPA: Epadel, randomized trial for evaluation in secondary prevention efficacy of combination therapy-statin and eicosapentaenoic acid
RLP-C: Remnant-like particle cholesterol
TG: Triglyceride
VLDL: Very-low-density lipoprotein cholesterol
